# Use of speaking valve on preventing respiratory infections, in critical traqueostomized patients diagnosed of dysphagia secondary to artificial airway. edisval study

**DOI:** 10.1186/2197-425X-3-S1-A936

**Published:** 2015-10-01

**Authors:** A Fernández Carmona, M Arias Díaz, E Aguilar Alonso, I Macías Guarasa, P Martínez López, MA Díaz Castellanos, EDISVAL Group

**Affiliations:** Virgen de las Nieves Hospital, Granada, Spain; Intensive Care Unit, Hospital Santa Ana, Motril, Spain; Intensive Care Unit, Hospital Infanta Margarita, Cabra, Spain; Intensive Care Unit, Hospital Carlos Haya, Malaga, Spain; Intensive Care Unit, Hospital Virgen de la Victoria, Malaga, Spain

## Introduction

Available data shows that dysphagia rate, secondary to artificial airway and prolonged mechanical ventilation, in critical tracheostomized patients is high (50-83%). Dysphagia is directly related to bronchial aspirations and respiratory infections. The rate of respiratory infections on tracheostomized patients is also very high (Some series next 100%). The re-establishment of airway using speaking valves allow the rehabilitation and post-recovery of those disorders, as well as deglutition and phonatory system rehabilitation.

## Objectives

Determine the usefulness of speaking valve in preventing respiratory nosocomial infections in critical tracheostomized patients diagnosed of dysphagia secondary to artificial ariway.

## Methods

Pilot phase of randomized multicenter controlled clinical trial. From September 2014 until December 2014, the use of speaking valve was randomized during mechanical ventilation weaning-decannulation phase in critical patients over 18 years, tracheostomized at ICU, diagnosed of dysphagia secondary to artificial airway. This study was carried out of simultaneous form in 7 units of intensive care including centers of the first, second and third level.

## Results

27 patients were included, 8 of which were excluded of the record and later analysis because of problems of protocols fulfillment. 19 included patients presented an average of 60,46 years of age, and initial APACHEII 19,07. The average time of mechanical ventilation was 23,6 days. After ramdomisation 11 patients were treated with speaking valve (SVgroup), and 8 by identical protocol without speaking valve (decannulation protocol DPgroup). The incident of infections in the SVgroup was 18,18% (2/11) and 37,5% (3/8) in the DPgroup. Infections in the SVgroup were registered like tracheobronquitis while in DPgroup as pneumonia. It is important to indicate that all the patients included in this study remained under absolute diet. Decannulation time average was 4,4 days in the SVgroup and 6 days in the DPgroup PD. Patients were decannulated when they fulfilled strict criteria (in both groups) including disphagia normalization. The mortality was 0 in the SVgroup and 3 in DPgroup.

There have not been registered side effects of the speaking valve use. After detecting mistakes we have proposed measures to avoid problems of follow-up of protocols. The incorporation of the SV in the different implied units has been easy and very well received.

## Conclusions

Incident of respiratory infectious complications and mortality was minor in the SVgroup. In the following months it will continue the experimental phase of EDISVAL study in order to know if these differences are attributable to speaking valve use.
